# Sand Fly Fauna and Prevalence of *Leishmania* spp. in a Newly Investigated Area of Northern Italy: Emerging Epidemiological Scenarios?

**DOI:** 10.1155/tbed/4426385

**Published:** 2025-07-21

**Authors:** Alessandro Alvaro, Giulia Maria Cattaneo, Fabio Bigoni, Lorenzo Sanchez-Ruiz, Jairo Alfonso Mendoza-Roldan, Domenico Otranto, Ilaria Varotto-Boccazzi, Paolo Gabrieli, Claudio Bandi, Sara Epis

**Affiliations:** ^1^Department of Biosciences, University of Milan 20133, Milan, Italy; ^2^Department of Veterinary Medicine and Animal Science, University of Milan 26900, Lodi, Italy; ^3^Department of Veterinary Medicine, University of Bari 70010, Valenzano, Italy; ^4^Department of Veterinary Clinical Sciences, City University of Hong Kong, Hong Kong, China

## Abstract

Since the 1990s, cases of canine leishmaniasis (CanL) due to *Leishmania infantum* have risen in northern Italy due to dog translocation and movement as well as for climate-driven sand fly population growth. In this geographical region, for a long time regarded as non-endemic for CanL, *L. infantum* is generally transmitted by the sand flies of the genus *Phlebotomus*. Other sand flies, such as *Sergentomyia minuta*, have been less investigated, also because they were only considered as vectors of the nonpathogenic herpetophilic *Leishmania tarentolae*. Our study investigates sand fly species composition and *Leishmania* spp. prevalence in hilly areas of northern Italy, in the Bergamo district. Sand flies were sampled with both sticky and light traps. Common wall lizards *Podarcis muralis* have been captured in the same areas, for the collection of biological samples: blood, feces, tissues, and gut-associated secretions. Sand flies were identified morphologically (males) and molecularly (females). The collected samples were tested for the presence of *Leishmania* spp. via conventional and digital droplet PCR. Our study reports the presence of *Phlebotomus perniciosus*, *Phlebotomus neglectus*, and *S. minuta* in the investigated area. We report the first detection of *L. tarentolae*, a *Sauroleishmania* species, in northern Italy, in both sand fly vectors (*S. minuta*, *P. perniciosus*, and *P. neglectus*) and reptile hosts. Additionally, *L. infantum* DNA was detected, for the first time, in sand flies and reptiles in the district, spatially overlapping with previously reported local CanL cases. Our study reported the presence of three sand fly species in the Bergamo district, a *Sauroleishmania* species (the first record in northern Italy), and the occurrence of *L. infantum*. We emphasize the importance of including both *S. minuta* sand flies and synanthropic reptiles in leishmaniasis screenings for a better understanding of the epidemiology of the disease.

## 1. Introduction

Phlebotomine sand flies (Diptera: Psychodidae) are small hematophagous insects of medical and veterinary importance, as they are the vectors of many viral, bacterial, and parasitic agents, such as those of the genus *Leishmania* (Kinetoplastida: Trypanosomatidae), the etiological agents of leishmaniases. These diseases pose a major public health concern in several regions worldwide, especially in the tropics and the subtropics, and are also endemic to the Mediterranean area [1]. In Italy, canine leishmaniasis (CanL) and the human cutaneous (CL) and visceral (VL) forms of the disease are caused by *Leishmania infantum* Nicolle, 1908 [2, 3]. In this country, sand flies of the species *Phlebotomus perniciosus* Newstead, 1911, and *Phlebotomus perfiliewi* Parrot, 1930, are considered the main vectors of *L. infantum* [4, 5]. *Phlebotomus neglectus* Tonnoir, 1921, and *Phlebotomus ariasi* Tonnoir, 1921, are also present in Italy, and have been recognized as vectors of *L. infantum* in other parts of Europe [6–9].

Since the 1990s, both human leishmaniases and CanL, traditionally considered restricted to southern and central Italy, have incremented their diffusion and their overall prevalence and incidence [10–14]. Cases of both human CL and VL, as well as CanL cases, have then been reported in the northern regions of the country [14–17]. Indeed, autochthonous cases of canine and human *Leishmania* infections have been recorded from all the northern Italian regions [14, 15].

The above mentioned changes in the epidemiological scenarios have been attributed, among others, to the traveling or relocation of infected dogs coming from hyperendemic areas [17] and the expansion of sand fly populations into new territories, likely as a consequence of climatic and environmental changes [16]. In this context, the increased density of *P. perniciosus* and *P. neglectus* populations northward has been associated with the emergence of autochthonous leishmaniases cases [18]. Other sand fly species, such as *P. perfiliewi*, *P. ariasi*, *Phlebotomus mascitti* Grassi, 1908, *Phlebotomus papatasi* (Scopoli, 1786), and *Sergentomyia minuta* (Rondani, 1843) have also been reported in northern Italy [13, 19–22]. The herpetophilic *S. minuta* is one of the most abundant and widespread species in the Mediterranean area, but it is normally not included in *Leishmania* spp. epidemiological studies, as it is regarded as the vector of the reptile-associated *Leishmania* (*Sauroleishmania*) *tarentolae* Wenyon, 1921. Indeed, *L. tarentolae*, a parasite that has been detected in both sand flies and reptiles in the Mediterranean area, is regarded as nonpathogenic to mammals [4, 23–25]. Notably, experimental evidence suggests that these parasites polarize the immune response towards the Th1 side, which might ensure protection to the pathogenic *Leishmania donovani* (Laveran and Mesnil, 1903) [26]. These observations have caught the attention of researchers, who are currently investigating *L. tarentolae* for the development of novel anti-*Leishmania* vaccines [27–29]. To date, the ecology and epidemiology of this parasite remains poorly understood, and little is known on the potential pathogenicity of *L. tarentolae* to mammals and other related saurian-associated *Leishmania* parasites. For example, some *L. tarentolae* strains can cause transient infections in laboratory conditions, and one case of systemic spread of *L. tarentolae* has been described in a human mummy [30, 31]. *Leishmania (Sauroleishmania) adleri* Heisch, 1958, a lizard-associated parasite from sub-Saharan Africa related to *L. tarentolae*, has been reported to cause infections in rodents [32] and humans [33]. Moreover, sequences of an unclassified *Leishmania* species detected in CL and VL cases in China showed a close relationship to *L. tarentolae*, suggesting that a similar parasite may be involved in the disease [34, 35].

Furthermore, other studies have indicated the presence of *L. tarentolae* DNA in dogs, humans, and in sand flies of the species *P. perniciosus* in central and southern Italy [23, 36]. The detection of *L. tarentolae* genetic material in *P. perniciosus* is in accordance with the fact that *L. tarentolae* has been reported to develop in *Phlebotomus* sand flies under laboratory conditions [37]. Moreover, laboratory-maintained *S. minuta* specimens readily bite humans [38], while wild *S. minuta* have been reported to feed on humans and other mammals in Spain and Italy [39, 40]. On the other hand, *L. infantum* DNA has been detected in *S. minuta* collected in the field [36]. The two parasites and their corresponding vectors can be found in sympatry [24, 36], and coinfection by both *Leishmania* species has been observed serologically in dogs and molecularly in sand flies [24].

*Leishmania* spp. DNA has been detected in synanthropic reptiles, with *L. tarentolae* infections reported from different species of lizards and snakes in Italy. Reptiles found positive to *L. tarentolae* belonged to the species *Tarentola mauritanica* (Linnaeus, 1758), *Podarcis filfolensis* (Bedriaga, 1876), *Podarcis siculus* (Rafinesque-Schmaltz, 1810), *Elaphe quatuorlineata* (Bonnaterre, 1790), and *Hierophis viridiflavus* (Lacépède, 1789) [25, 41]. Moreover, in southern Italy, *L. infantum* DNA has been detected in lizards and geckos of the species *Hemidactylus turcicus* (Linnaeus, 1758), *T. mauritanica*, and *P. siculus* [25], and that of *L. donovani* and *Leishmania turanica* Strelkova and colleagues 1990 in snakes and lizards from China [35, 42, 43]. Based on the current evidence, understudied herpetofauna and sand fly species may play a significant role in the emergence of *Leishmania* parasites in new regions and their maintenance in endemic areas. Given the lack of information on the occurrence of *Leishmania* spp. in the common wall lizard *Podarcis muralis* (Laurenti, 1768), the most common synanthropic reptile in northern Italy, and in *S. minuta*, we investigated the sand fly species composition and the prevalence of *Leishmania* spp. in a northern Italian district, in both sand fly vectors and synanthropic reptile hosts.

## 2. Materials and Methods

### 2.1. Study Sites and Sand Flies Sampling

**Figure 1:**
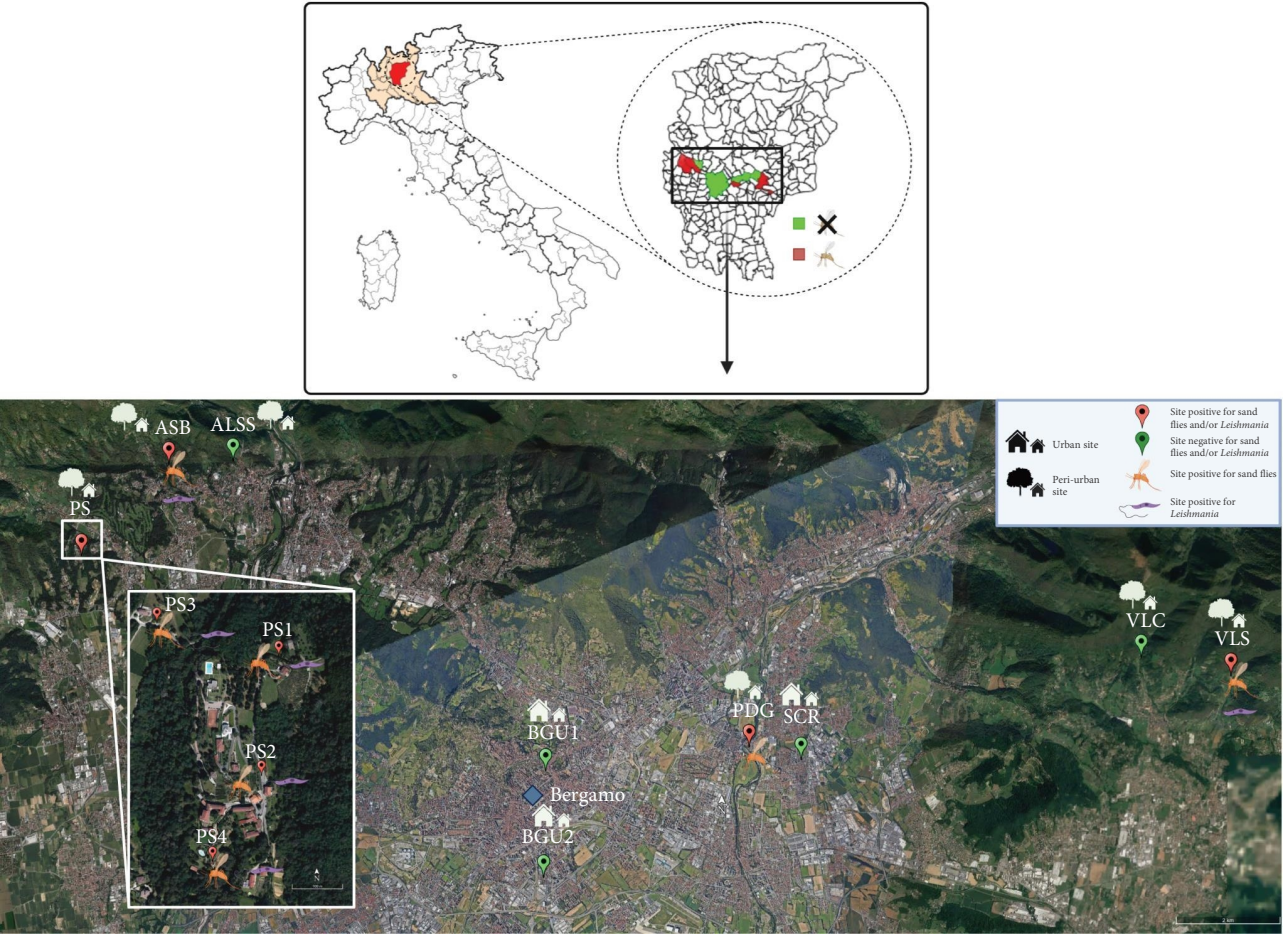
Location within Italy and satellite image of the sites in which sand flies monitoring was carried out.

Sand fly collections were carried out in nine peri-urban sites and three urban sites of the Bergamo district (Lombardy Region, Italy) (Figure 1). These sites comprised a variety of environmental conditions: six of the peri-urban sites were in hilly areas, characterized by the presence of deciduous vegetation, bushes, rocks, and stone walls. Livestock, housed in shelters, and domestic animals were present, as well as synanthropic reptiles. The other three peri-urban sites were represented by a private kennel, a private chicken coop, and a forest margin. The urban sites were in private gardens, which featured stone walls and in which synanthropic reptiles were present. A full description of the collection sites is included in Table 1.

Sand fly samplings were conducted during the sand fly activity season, between July and October 2022 and between June and October 2023. In the 2022 season, the sampling was conducted in five peri-urban sites and one urban site. Three out of five sites were again sampled in 2023, with the addition of four peri-urban and two urban sites. Sand flies were collected by setting traps one night per week. If no sand fly was collected after three sampling sessions, the site was considered negative. If a site resulted positive to the presence of sand flies, the sampling continued until there were three consecutive negative captures.

**Figure 2:**
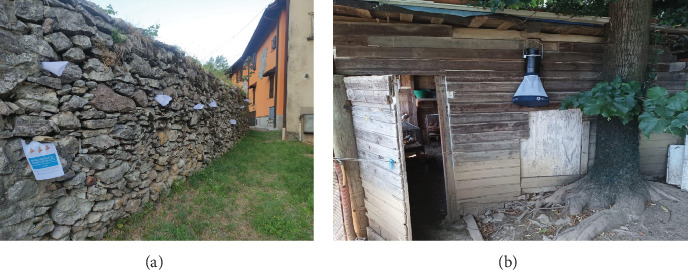
Representative sites in which sticky traps were employed (a) and light traps were positioned (b) for the collection of sand flies.

The sampling was realized employing both sticky traps (A4 papers coated with castor oil), and BG-Pro light traps in CDC configurations (Biogents, Regensburg, Germany). In this configuration, the light traps featured an LED light employed to attract insects. The light traps were also equipped with a CO_2_ source, the BG-Lure (Biogents, Regensburg, Germany). The sticky traps were placed inside holes and crevices of stone walls and rocks, while light traps were settled inside animal shelters or near putative sand fly resting places (Figure 2). Both types of traps were set in the sites before sunset and retrieved the morning after. Sand flies were collected from sticky traps after being macroscopically recognized and then stored in 100% ethanol. The content of the light trap was put in a cool box. The collected insects were taken to the EntoPar laboratory of the University of Milan.

An earlier version of the study, including the sampling design and preliminary data, was presented as part of the first author's PhD thesis, submitted to the University of Milan [44].

### 2.2. Sand Fly Species Identification

Once in the lab, the light traps content was put in 100% ethanol, and sand flies were isolated from other collected insects, following observation under a Leica M50 stereomicroscope (Leica Microsystems, Germany). Male sand flies were identified on the basis of the genital structure, which was dissected under the stereomicroscope and then observed under a Leica DM1000 microscope (Leica Microsystems, Germany). Species determination was then carried out following a reference morphological key [45].

Total genomic DNA was individually extracted from the whole body of each female sand fly, with no pooling of specimens. In particular, female sand flies were subjected to total DNA extraction using the DNeasy Blood & Tissue kit (QIAGEN, Hilden, Germany) following the manufacturer protocol specific for insects.

Female sand fly species identification was performed following a polymerase chain reaction-restriction fragment length polymorphisms (PCR-RFLP) protocol [46]. DNA was extracted from morphologically determined male sand flies and used as a positive control in the PCR-RFLP experiments.

### 2.3. Reptile Capturing and Sampling

Synanthropic reptiles were captured by hand or using nets around the sand fly sampling sites.

From reptiles, we collected the following six categories of biological samples: blood, feces, saliva, cloacal secretions, and tail tissue, and we performed blood smears. Blood was obtained via venipuncture of the ventral coccygeal vein or via cardiocentesis. A drop of blood was used to prepare a blood smear. Feces were collected after voluntary expulsion by the animal or by gently compressing their abdomen. Oral and cloacal samples were collected using cotton swabs. If dropped voluntarily during manipulation, the tail was collected and stored in 70% ethanol. After collection, all the samples were stored immediately in a cool box. After sampling, the animals were placed in a fauna box and monitored for approximately 1 h, after which they were released where they were captured. Dead animals were collected, if encountered, and placed in 70% ethanol. Once in the laboratory, all the samples were stored at −20°C.

Blood smears were stained with Giemsa and then observed under a Leica DM1000 microscope for *Leishmania* amastigotes and promastigotes detection. DNA was extracted from all the collected blood samples using the DNeasy Blood & Tissue kit following the manufacturer's protocol for animal blood and feces, respectively.

From 10 lizards, it was possible to collect at least five out of the six sample categories mentioned above. Therefore, DNA extraction was also realized from tails as well as from oral and cloacal swabs. The extraction was performed using the DNeasy Blood & Tissue kit, following the manufacturer's protocol specific to animal tissues for the tails, and the protocol for buccal swabs for the oral and cloacal swab samples.

### 2.4. Detection of *Leishmania* spp.

Female sand flies and reptile samples were screened via PCR to detect *Leishmania* spp. DNA using pan-*Leishmania* LITSR/L5.8S primers targeting a ~300 bp region of the internal transcribed spacer 1 (ITS-1) region [47]. PCR was performed in a SimpliAmp thermal cycler (Applied Biosystems, Waltham, MA). PCR reactions were carried out in a final volume of 25 μL, consisting of 5 μL of 5X GoTaq Reaction Buffer (Promega Corporation, Madison, WI), 2.5 μL of nucleotides, 0.125 μL of GoTaq G2 DNA Polymerase (Promega Corporation, Madison, WI), 1.25 of both forward and reverse primers at 10 μM concentrations, 12.875 μL of Milli-Q water, and 2 μL of DNA. *L. tarentolae* Lt-P10 strain (Jena Bioscience, Jena, Germany) and *L. infantum* MHOM/TN/80/IPT-1 strain (WHO) DNA extracted from laboratory-maintained clones was used as a positive controls, while Milli-Q water was added instead of DNA in the negative control. The PCR thermal protocol consisted of an initial denaturation step at 98°C for 3 min, followed by 40 cycles of denaturation at 95°C for 15 s, annealing at 55°C for 15 s and annealing at 72°C for 15 s, and a final elongation step at 72°C for 10 min.

PCR products were visualized in a 1.5% agarose gel, and bands with an adequate weight were purified using the Monarch DNA Gel Extraction Kit (New England BioLabs, Ipswich, MA). The purified products were sequences in both directions with Sanger technology (Eurofins Genomics GmbH, Konstanz, Germany).

### 2.5. Droplet Digital PCR (ddPCR) Screening of Engorged Sand Flies and Synanthropic Reptile's Blood for *L. tarentolae* and *L. infantum*

The engorged sand flies and the blood collected from synanthropic reptiles were screened via ddPCR for the simultaneous detection of *L. tarentolae* and *L. infantum* kinetoplast DNA (kDNA), following the protocol described in Alvaro et al. [48]. This method employs a single primer pair to amplify a 53 bp kDNA fragment from both *L. tarentolae* and *L. infantum*, and two species-specific TaqMan probes enabling their differentiation. During the assay validation, standard curves were generated by spiking dog blood with DNA extracted from known numbers of cells of *L. tarentolae* and *L. infantum* reference strains. This allowed for an estimation of the number of parasite cells infecting the tested sand flies and reptile blood samples, by comparing their ddPCR target concentrations with the ones resulting from the above-mentioned standard curves.

### 2.6. Engorged Sand Flies Blood Meal Determination

Blood meals of engorged female sand flies were molecularly determined by PCR targeting a fragment (~350 bp) of the vertebrate cytochrome b (cyt b) mitochondrial gene, using universal vertebrate primers cyt bb1/cyt bb2, as in Radrova et al. [49]. The PCR mix, the electrophoresis conditions, and the product purification procedures were the same as those adopted during *Leishmania* spp. detecting experiments of sand flies.

### 2.7. Phylogenetic Analysis

The sequences generated in the present study were subjected to an nBLAST search. We downloaded the resulting hits (consisting mostly of *Leishmania* sp. sequences), along with all *L. tarentolae* hits, as well as Chinese *Leishmania* and *L. adleri* sequences. One reference sequence each for *Leishmania tropica* Wright, 1903, *Leishmania major* Yakimoff & Schokhor, 1914, *L. infantum*, and *L. donovani* was included as an outgroup. The abovementioned sequences accession numbers are reported in Table S1. All the sequences were aligned with MUSCLE, version 3.8.425 [50], within the Aliview software [51]. For the Maximum Likelihood (ML) phylogenetic analysis, all the BLAST hits, along with the selected outgroups, were analyzed using MEGA, version 11.0.10 [52]. The K2 + G substitution model was chosen after a BIC-score analysis of nucleotide substitution models. A number of 1000 bootstrap replicates were computed. The resulting phylogenetic tree was edited using the TreeViewer software [53].

## 3. Results

### 3.1. Sand Fly Collection

Sand flies were found in 7/12 sites, and a total of 679 specimens were collected during the study (Table 2). Sand flies were identified as *P. perniciosus*, *P. neglectus*, and *S. minuta*. A total of 170 *P. perniciosus* (122 males and 48 females), 65 *P. neglectus* (53 males and 12 females), and 444 *S. minuta* (246 males and 198 females) were collected. The numbers of collected sand flies for each site are indicated in Table 2.

### 3.2. Reptile Samples Collection

In total, 54 synanthropic specimens of *P. muralis* were captured between 2022 and 2023 in the peri-urban “PS1”, “PS2”, “PS3”, and “PDG” sites. A total of 35 blood samples, 41 fecal samples, 10 salivary swabs, 10 cloacal swabs, and 10 tissue samples were collected. A number of 35 blood smears were performed. The collection of at least four out of the five acquirable kinds of samples from the same specimen was possible in 10 lizards.

### 3.3. PCR Detection of *Leishmania* spp. in Sand Flies and Synanthropic Reptiles

We found *Leishmania* spp. positive sand flies at all sites where sand flies were collected, except for “PDG”, where we collected only a single male. Considering all the collected females, 22.48% (58/678) resulted positive to *Leishmania* spp. by ITS-1-targeted PCR. Specifically, 31.25% (15/48) of *P. perniciosus*, 16.67% (2/12) of *P. neglectus*, and 20.71% (41/198) of *S. minuta* females resulted positive to *Leishmania* spp. (Table 3). For reptile samples, 80% (28/35) of blood samples, and 31.71% (13/41) of fecal samples (considering all the sites of reptile sampling) resulted positive to *Leishmania* spp. DNA after the ITS-1targeted PCR. In general, 62.96% (34/54) of collected individuals resulted positive to *Leishmania* spp. at least for blood or faeces. Regarding lizards from which both blood and faeces were collected, 27.78% (5/18) resulted positive to *Leishmania* spp. in both sample categories.

A subset was constituted by lizards from which it was possible to collect at least four out of five kinds of samples. In this subset, all the blood samples and tissue samples resulted positive to *Leishmania* spp. DNA after PCR, while 9/10 fecal and salivary samples and 8/10 cloacal samples resulted positive to *Leishmania* spp. DNA after PCR. A number of 6/10 individuals resulted positive to *Leishmania* spp. DNA after PCR.

A total of 50 ITS-1 sequences were generated from the PCR products, 35 obtained from sand flies and 15 from *P. muralis* lizards (Table S2). Moreover, one sequence was generated from the Lt-P10 strain (Jena Bioscience, Jena, Germany), maintained in the EntoPar laboratory.

### 3.4. ddPCR Detection of *Leishmania tarentolae* and *Leishmania infantum* in Engorged Sand Flies and Synanthropic Reptiles Blood

Following ddPCR screening of the engorged sand flies for *L. tarentolae*, 91.89% (68/74) exhibited a signal exceeding the threshold of four positive droplets, as reported by Alvaro et al. [48]. Based on the ddPCR assay protocol cited above, 4.41% of sand flies were estimated to harbor between 25 and 250 *L. tarentolae* cells, 25% contained five to 25 cells, 16.18% had one to five cells, and 54.41% carried fewer than one *L. tarentolae* cell. The frequency of *L. infantum* detection was 13.33% (2/15) in *P. perniciosus*, 66.67% (2/3) in *P. neglectus*, and 5.45% (3/55) in *S. minuta* engorged females. Overall, *L. infantum* was detected 9.46% (7/74) of the engorged females. In all the positive samples, the estimated parasite load was less than one *L*. *infantum* cell. Six out of seven sand flies positive to *L. infantum* were also positive to *L. tarentolae*, while one resulted positive to *L. infantum* only.

Regarding reptiles, 97.14% of the blood samples (34/35) exceeded the positivity threshold after ddPCR screening for *L. tarentolae*. Of these samples, 2.94% were estimated to harbor between 5 and 25 *L. tarentolae* cells, 29.41% contained 1–2.5 cells, and 67.65% had 1–5 *L. tarentolae* cells. After ddPCR screening for *L. infantum*, 8.57% of blood samples (3/35) resulted positive. As for sand flies, the estimated *L. infantum* load was less than one parasite cell in every positive sample.

### 3.5. Engorged Sand Flies Blood Meal Determination

Host DNA was amplified in 36.11% (26/72) of the engorged female sand flies. Of these, 42.6% (23/54) *S. minuta* females fed on *P. muralis*, nine of which resulted positive for *Leishmania* spp. after PCR; 13.33% (2/15) of *P. perniciosus* females fed on sheep, while one fed on a horse. None of the three *P. perniciosus* resulted positive for *Leishmania* spp. after PCR. Host DNA wasn't successful for any of the three engorged female *P. neglectus*, although one resulted positive for *Leishmania* spp. after PCR.

### 3.6. Reptiles Blood Smear Observations

**Figure 3:**
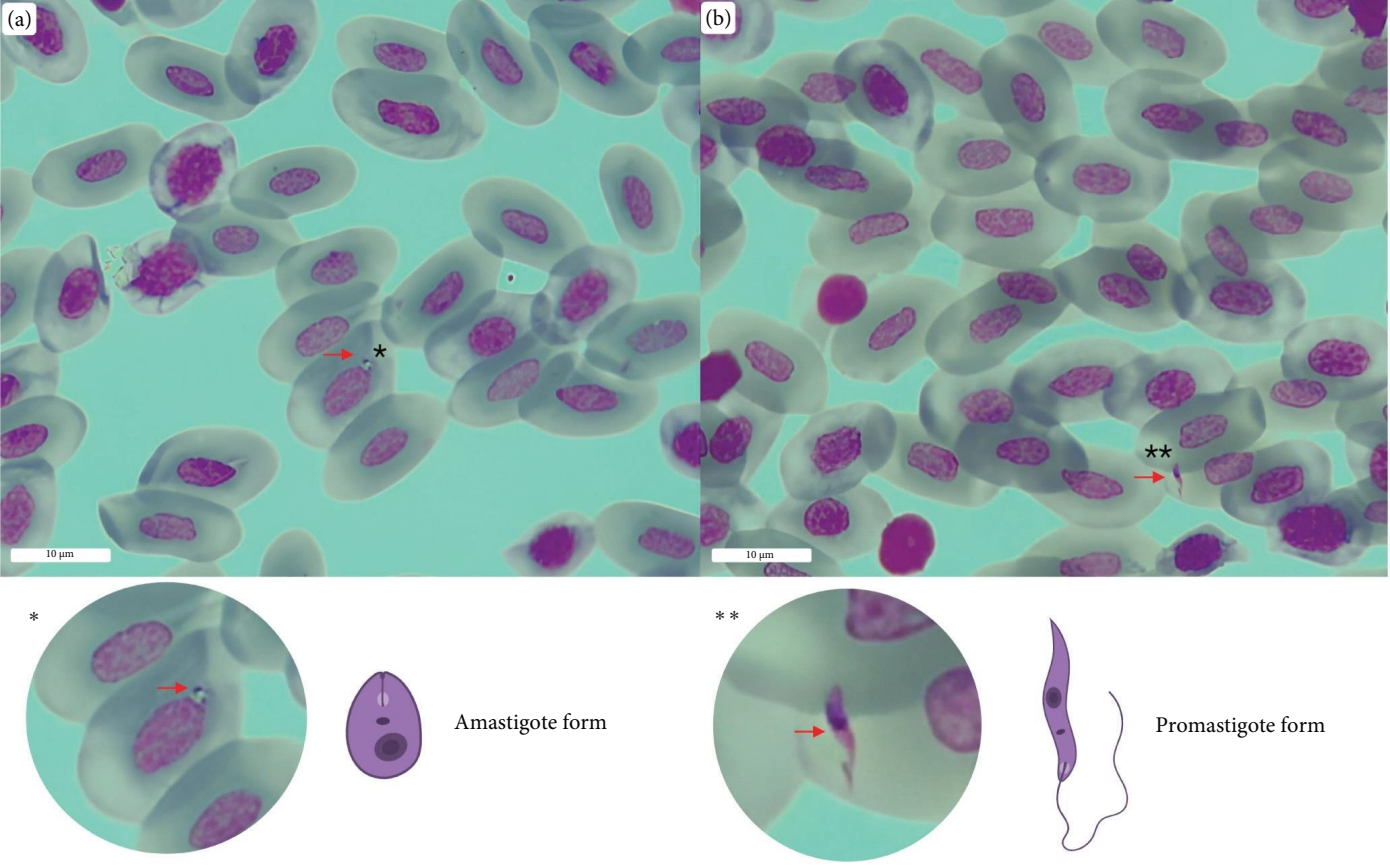
*Leishmania*-like parasites as observed in a blood smear of a *Podarcis muralis* lizard positive to *Leishmania tarentolae* after ITS-1 targeted pan-Leishmania PCR. (a) An amastigote-like form is seen inside erythrocytes (indicated by the red arrow). (b) A promastigote-like form is also visible (red arrow). *⁣*^*∗*^ and *⁣*^*∗∗*^ indicate parasites amastigote-like and promastigote-like forms, respectively, within the blood smears. The scale bar indicates a measurement of 10 µm.

The observation of both amastigote and promastigote-like forms was reported in one out of 35 blood smears. The blood smear belonged to a *P. muralis* specimen for which *Leishmania spp*. DNA was molecularly detected in every sample category (Figure 3).

### 3.7. Phylogenetic Analysis

The best BLAST hits of the sequences generated in our study are entries from organisms indicated as *Leishmania* sp., generated from *S. minuta* from Spain (accession numbers: MK567803.1, MK567795.1, LC216356.1, MK567798.1, MK567806.1, LC216368.1, MK567786.1, and MK567800), and a domestic dog from Iran (Accession Number: MT302155.1). All the above entries share high levels of similarity (97.9%–99.7%), and the ones derived from *S. minuta* have been indicated as related to *L. tarentolae* [40, 54]. Similarly, the percentage of identity of the sequences we generate with *L. tarentolae*, as determined through BLAST search, varied from 91.49% to 98.50%. In contrast, the sequences generated in our study, as well as those mentioned above, showed a lower percentage identity with *L. adleri* (generally <90%) compared to *L. tarentolae*. We therefore anticipate that there is a group of entries in the data bases that are indicated as *Leishmania* sp. but could possibly be reclassified as *L. tarentolae* (see below and discussion), and that show high similarity with the sequences generated in this study.

Following the phylogenetic analysis, the bootstrap consensus tree (Figure S1) revealed a lack of genetic structuring among the *Sauroleishmania* sequences, which include the ones generated in this study and the BLAST hits included in the analysis, as well as the *L. adleri* sequences, resulting in most of the nodes in being unresolved. Nevertheless, the *L. adleri* sequences were clearly distinct and grouped into a monophyletic clade (with 98% bootstrap support). The outgroup, that is the sequences of the subgenus *Leishmania*, formed a monophyletic clade (bootstrap support: 99%).

**Figure 4:**
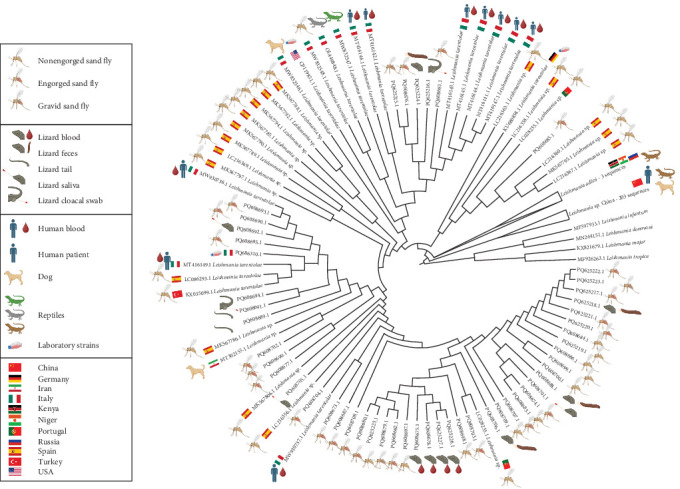
Tree with the highest log likelihood, according to the K2 + G nucleotide substitution model, realized with MEGA software. The substitution model was chosen after a BIC-score analysis. The origin of the sequences is indicated by icons, explained by the legend.

In the tree with the ML (Figure 4), all the sequences generated in this study formed a monophyletic clade with GenBank entries belonging to *L. tarentolae* and with other entries in GenBank deposited as *Leishmania* sp., in agreement with the results of BLAST search (see above). As for GenBank entries deposited as *L. tarentolae*, these originated from sand flies (*S. minuta*, *Sergentomyia dentata* (Sinton, 1933), reptiles (*P. siculus*, *T. mauritanica*), and mammalian hosts (human and domestic dog) coming from southern Italy, Spain, and Turkey (Table S1). Sequences of the laboratory-maintained *L. tarentolae* strains P10 (among them, a sequence generated in the present study) and M2 cluster in this clade. These cultured isolates are kept in Italy, Iran, and the United States.

The *Leishmania* sp. sequences that clustered in this clade come from *S. minuta* screened in Spain and Portugal. The three available *L. adleri* sequences formed a monophyletic clade sister to the *L. tarentolae* plus *Leishmania* sp. clade, while the Chinese *Leishmania* sp. sequences formed a group basal to the clade composed by *L. tarentolae* plus *Leishmania* sp. and the *L. adleri* clade.

## 4. Discussion

The data from the present study provides the first evidence of the existence of an autochthonous cycle of *Sauroleishmania* in northern Italy, being the parasite DNA detected in both sand flies and in common wall lizards hosts. Given the significant sequence similarity of the sequences generated in our study with those related to *L. tarentolae*, along with their phylogenetic placement, we can confidently conclude that the northern Italian sequences presented here belong to a species of the subgenus *Sauroleishmania*. Moreover, since the sequences generated in our study don't segregate from the sequences of *L. tarentolae* after phylogenetic analyses, they may be considered as belonging to the *L. tarentolae* species, also after taking into account their geographic origin and the host from which they derive. In addition, we also suggest that the *Leishmania* sp. entries, that we downloaded from GeneBank to perform phylogenetic analyses, should be attributed to *L. tarentolae*, for the same reasons. Indeed, another piece of evidence supporting this finding is that these sequences primarily originated from *S. minuta*, the confirmed vector of the parasite, sourced from countries where *L. tarentolae* is endemic.

A finer-scale and bootstrapped-supported structuring within the *Sauroleishmania* clade could not be resolved due to the high sequence similarity observed across the ITS-1 region (Figure S1) Therefore, other molecular markers should be employed to uncover potential intraclade divergence patterns that are not detectable with ITS-1 alone.

As expected, we detected *L. tarentolae* in *S. minuta*, the recognized sand fly vector of reptile-associated *Leishmania*. The overall *L. tarentolae* prevalence in sand flies in this study, considering all sites and species, was 20.71% based on ITS-1-targeted PCR. This finding aligns with the results of Gonzalez et al. [40] in Spain, where the parasite referred to as a *Leishmania sp*. in their publication had an 18% prevalence in 377 tested *S. minuta* sand flies using the same molecular assay. In Portugal, however, the prevalence of the parasite (there identified as *Leishmania* sp.) was considerably lower, with only two out of 1867 *S. minuta* positive to *L. tarentolae* DNA by amplification of the same target [55].In southern Italy, a 12.62% prevalence of *L. tarentolae* DNA was recorded in the same sand fly species by qPCR [24].

The ddPCR results showed a higher prevalence of *L. tarentolae* (97.14%) than the one obtained after PCR in the tested engorged sand flies and revealed the occurrence of *L. infantum* DNA in the same sand flies. This could be attributed to the greater sensitivity and specificity of the ddPCR assay, which targets a conserved region of the kDNA minicircles, compared to the conventional PCR that focuses on the ITS-1 region. In fact, the ddPCR was able to detect even very low concentrations of *Leishmania* DNA, corresponding just to the parsasites “molecular signatures”. A molecular signature detected from less than a single *Leishmania* cell may arise under several scenarios, such as (i) early-stage infection, where the pathogen load is still minimal, and the infection has not yet fully established itself; (ii) environmental acquisition, where the molecular signature might be acquired due to proximity of and mere contact between infected sand flies, such as during prediuresis events, rather than reflecting an actual infection in the host, and (iii) residual presence from past infection, where the signature could result from a previous infection that occurred long ago, leaving behind only traces of molecular evidence without any active infection.

The *S. minuta* collected during our work showed a pure herpetophilic behavior, as the vertebrate DNA amplified from them belonged to *P. muralis*, with no evidence of feeding on mammals and humans, as reported in other studies [24, 39, 40, 55]. We also detected *L. tarentolae* in *P. perniciosus*, confirming the previous findings of studies carried out in southern Italy [24, 36] and in accordance with vector competence studies on this species [37]. The prevalence of *L. tarentolae* in the tested *P. perniciosus*, assessed with conventional PCR, was 31.25% considering all the sites, the highest recorded to date. In other studies, in southern Italy, prevalence of *L. tarentolae* in *P. perniciosus* was reported to be less than 10% [24, 56]. Moreover, we detected *L. tarentolae* DNA for the first time in *P. neglectus*, with an overall prevalence of 16.67%. However, *P. perniciosus* fed on horse and sheep and not on *P. muralis*, while it wasn't possible to amplify host DNA from engorged *P. neglectus* females. It may be possible that the *P. perniciosus* and *P. neglectus* of the sampled populations are characterized by an opportunistic feeding behavior, feeding on the blood of both mammals and reptiles. Strong evidence for this scenario is the high prevalence of *L. tarentolae* DNA detected in *P. perniciosus* and *P. neglectus*. Both the *P. perniciosus* and the *P. neglectus* specimens of the studied areas could be competent in the transmission of *L. tarentolae* to both reptiles and mammals. More sand fly samples are needed in order to elucidate the feeding preferences of sand flies in the study area. Moreover, samples of mammals living in the sand fly areas need to be screened to better comprehend the biological cycle of the *L. tarentolae* parasites detected in the study.

In the peri-urban “VLS” site, specimens of both *P. perniciosus* and *P. neglectus* were found positive to *L. tarentolae* DNA, with only three *Leishmania* spp. negative *S. minuta* females collected in the site. This may be due to the fact that in the “VLS” site sand flies were captured only with a light trap, which may not be attractive for *S. minuta*. Evidence for this hypothesis comes from the sampling results of the “PS1” site, where sticky traps were used to capture sand flies in 2022, and in 2023 they were alternate with light traps. No *S. minuta* specimens were collected using light traps. This may explain the limited number of *S. minuta* collected with light traps in the “VLS” site.

Furthermore, our study reports the first detection of *L. tarentolae* DNA in *P. muralis*; the high prevalence uncovered in blood (>70%) and fecal samples (>30%) suggests that *P. muralis* is an important reservoir for *L. tarentolae*. The prevalence in the analyzed *P. muralis* population is higher than that recorded for reptiles collected in southern Italy [25] and is comparable with prevalence values recorded in China [35]. Despite this high prevalence of *L. tarentolae* in the lizard's blood, as revealed by PCR, we observed promastigote- and amastigote-like cells only in one blood smear, out of the examined 35 samples. Interestingly, we detected *L. tarentoale* DNA also in tissue samples and in salivary and cloacal swabs. The detection of *L. tarentolae* DNA in both blood and feces, and in other gut-associated secretions (saliva and cloacal secretions), is congruent with the idea that transmission of *Leishmania* spp. in reptiles might occur through: (i) the bite of infected sand flies; (ii) after the ingestion of sand flies; (iii) following the exposure to prediuresis secretions produced by sand flies [57]. The high prevalence of parasite DNA in lizard blood combined with the finding that *S. minuta* feeds on *P. muralis* indicates that inoculation of *L. tarentolae* via sand fly bite is likely a major mode of transmission. This hypothesis is also heavily supported by the results obtained after screening the samples with a more sensitive ddPCR assay, in which >90% of the blood samples resulted positive to *L. tarentolae*. The fact that only one blood smear revealed the presence of *Leishmania*-like cells in erythrocytes may be due to the ability of the parasite to colonize internal organs in the reptile host. This is in accordance with the detection of *L. tarentolae* DNA in organs of *P. siculus* in Southern Italy, although *L. tarentolae* was not detected in blood, feces and tails samples [24]. However, the detection of *L. tarentolae* DNA in feces and cloacal and oral swabs supports the possibility of a transmission of the parasite via ingestion by reptiles of infected sand flies. On the other hand, positivity of cloacal and oral swabs may also reflect the ability of *L. tarentolae* to visceralize in the reptile host, as in dogs positivity of conjunctival swab is associated with visceralization [58]. Finally, evidence supporting the transmission of *Sauroleishmania* parasites through the prediuresis of infected sand flies may be found in the positivity of tissue samples. These samples, which often harbor skin lesions, could serve as entry points for the parasites introduced via sand fly prediuresis secretions. Based on the evidence discussed above, we believe that the transmission of *L. tarentolae* through the bite of an infected sand fly is the most important and likely route.

We captured the lizards examined in this study in the same holes in which we collected sand flies with sticky traps. In addition, we also found shed lizard skins on the sticky traps. In this context, the sand flies could feed on the lizards resting inside the abovementioned holes during the evening hours, while the lizards could prey on sand flies during the morning and afternoon, when sand flies are inactive and stay inside the holes. Therefore, our results and observations are thus congruent with the existence of at least two modes of transmission of *Sauroleishmania* in reptiles; via sand fly bite, and after sand fly ingestion. This vector–host scenario, reflecting a close spatial relationship between sand flies and lizards, may also explain the high prevalence of *L. tarentolae* in lizard blood samples.

Numerous private properties present in the areas of sand fly sampling were characterized by the presence of dogs and cats. These pets may be exposed to *L. tarentolae* by being bitten by infected sand flies or by predating infected synanthropic lizards. Whether *Sauroleishmania* spp. in dogs and cats cause any pathology [30–33], or have some positive effect, such a stimulation of the immune response, that might be protective against canine and feline leishmaniasis [26], deserves further investigations [27]. On the other hand, since there is evidence that reptiles can host *Leishmania* spp. pathogenic to mammals [25, 43], predation on synanthropic reptiles may represent a risk factor for the acquisition of a *L. infantum* infection. Indeed, we detected DNA of *L. infantum* in the blood of the lizards of the sampled sites, following a ddPCR screening of the samples. The parasite DNA was found with a prevalence of 8.57% in *P. muralis* blood samples. This value is comparable with the 5.5% prevalence observed in *P. siculus* of southern Italy, although this result was obtained after testing samples of different tissues [24]. The concentrations of the parasitic DNA were very low and corresponded to less than one parasite cell. This is consistent with the following hypotheses: i) *L. infantum* may establish only transient infections in the lizards, which are not permissive reservoirs for the parasite; ii) *L. infantum* DNA might be inoculated to the lizards by *S. minuta* sand flies, which, despite having been found molecularly positive to *L. infantum* DNA in different reports, are still not regarded as a competent vectors of the parasite, and iii) blood may not be the ideal sample for *L. infantum* detection in lizards, as in the case of dogs [58]. *Leishmania infantum* is the causative agent of zoonotic VL leishmaniasis in mammals, with evidence also indicating its tropism for reptiles. The role of reptiles as reservoirs for *L. infantum* remains unclear; however, these animals may hold significance as “sentinels” for the presence of the parasite.

By employing the ddPCR method, we also report the detection of *L. infantum* DNA in engorged sand flies from the sites “PS1”, “PS2”, and “VLS”. This is, to the best of our knowledge, the first published report of the detection of this parasite in sand flies in the Bergamo district and in the whole Lombardy region. The finding of sand flies positive to *L. infantum* in the sampling sites is in accordance with the report of two cases of autochthonous CanL in the Imagna Valley [8]. *Leishmania infantum* DNA was detected in specimens belonging to all the three examined sand fly species. In *P. neglectus*, two out of three engorged females tested positive to this parasite. The prevalence of *L. infantum* in *P. perniciosus* was 13,33%, (2/15 positive sand flies), while in *S. minuta* was 5.45% (3/55 positive sand flies). The *L. infantum* prevalence values for *S. minuta* are higher than those reported for sand flies in southern Italy and Portugal [59, 60].

The detection of *L. infantum* in *P. perniciosus* is congruent to the fact that the species is a proven vector of the parasite [4]. Before our current report, *L. infantum* DNA has been detected in *P. neglectus* from southern Italy [53]. While there is evidence for the involvement of *P. neglectus* in the transmission of *L. infantum* in the Balkans [7], its actual role as a vector of this parasite is still to be confirmed. The finding of *L. infantum*-positive *S. minuta* is congruent with results from screening performed in central and southern Italy [24, 36]. The positivity also to *L. tarentolae* in six of the seven sand flies positive to *L. infantum* is in accordance with previous studies in southern Italy, in which positivity for the two parasites is reported in sand flies [24]. The positivity to *L. infantum* by ddPCR may be due, as discussed above, to the fact that the assay targets a conserved region of the minicircles of the kDNA, thus being more sensitive than the ITS-1-targeted conventional PCR. In fact, the concentrations of the detected parasitic DNA were very low. More samples, however, need to be collected and Sanger sequences must be generated to confirm the occurrence of this parasite in both sand flies and reptiles.

## 5. Conclusion

Our study contributes to the knowledge of the sand fly species composition of northern Italy, reporting the presence of *P. perniciosus*, *P. neglectus*, and *S. minuta* in the Bergamo district as well as the first report of *L. tarentolae* in northern Italy. We also report the occurrence of sand flies and reptiles positive to *L. infantum*, which is consistent with CanL cases recorded in the Bergamo district.

We emphasize that the inclusion of both *S. minuta* sand flies and synanthropic reptiles' samples in *Leishmania* spp. screenings might allow scientists to obtain a more comprehensive epidemiological picture and a better understanding of the dynamics of leishmaniasis in investigated areas.

## Figures and Tables

**Table 1:** Information on the sites in which sand flies collection was performed.

Name	Typology	Description	Municipality	Coordinates	Years of sampling	Trap/(s)
PS1	Peri-urban	Stonewall near livestock enclosure	Palazzago	45.740338, 9.556340	2022–2023	Sticky 2022/light 2023
PS2	Peri-urban	Stonewall	Palazzago	45.738119, 9.555963	2022–2023	Sticky
PS3	Peri-urban	Stonewall	Palazzago	45.740898, 9.553106	2023	Sticky
PS4	Peri-urban	Private kennel	Palazzago	45.736697, 9.554766	2023	Light
ASB	Peri-urban	Stonewall at wood margin	Almenno San Bartolomeo	45.752813, 9.575624	2022–2023	Sticky
ALSS	Peri-urban	Wood	Almenno San Salvatore	45.754995, 9.593900	2022	Sticky
VLS	Peri-urban	Wood margin	Trescore Balneario	45.717334, 9.843545	2023	Light
VLC	Peri-urban	Stonewall near livestock enclosure	Cenate Sopra	45.720935, 9.820993	2022	Sticky
PDG	Peri-Urban	Private chicken coop	Pedrengo	45.704915, 9.723497	2023	Light
BGU1	Urban	Private garden	Bergamo	45.700605, 9.672415	2022	Sticky
BGU2	Urban	Private garden	Bergamo	45.682070, 9.671917	2023	Light
SCR	Urban	Private garden	Scanzorosciate	45.702687, 9.736528	2023	Sticky

**Table 2:** Total number of sand flies collected and trapping method used for every site.

Site name	*P. perniciosus*			*P. neglectus*			*S. minuta*			TOT	Trap used
	M	F	TOT	M	F	TOT	M	F	TOT		
PS1	67	26	93	13	6	19	59	65	124	236	Sticky + light
PS2	2	2	4	8	2	10	159	106	265	279	Sticky
PS3	6	2	8	4	0	4	4	3	7	19	Sticky
PS4	10	1	11	4	0	4	0	0	0	15	Light
ASB	16	1	17	1	0	1	24	21	45	63	Sticky
VLS	21	16	37	22	4	26	0	3	3	66	Light
PDG	0	0	0	1	0	1	0	0	0	1	Light
TOT	122	48	170	53	12	65	246	198	444	679	—
ALSS	0	0	0	0	0	0	0	0	0	—	Sticky
VLC	0	0	0	0	0	0	0	0	0	—	Sticky
BGU1	0	0	0	0	0	0	0	0	0	—	Sticky
BGU2	0	0	0	0	0	0	0	0	0	—	Light
SCR	0	0	0	0	0	0	0	0	0	—	Sticky

**Table 3:** *Leishmania* spp. prevalence values in all female sand flies obtained after ITS-1 targeted conventional PCR.

Species	N. F sand flies	N. F Leish+	F Leish+ (%)
2022			
* Phlebotomus perniciosus*	12	1	8.33
* Phlebotomus neglectus*	2	0	0
* Sergentomyia minuta*	84	10	11.90
TOT	98	11	11.22
2023			
* Phlebotomus perniciosus*	36	14	38.89
* Phlebotomus neglectus*	10	2	20
* Sergentomyia minuta*	114	31	27.19
TOT	160	47	29.38
2022 + 2023			
* Phlebotomus perniciosus*	48	15	31.25
* Phlebotomus neglectus*	12	2	16.67
* Sergentomyia minuta*	198	41	20.71
TOT	258	58	22.48

## Data Availability

All the data produced during the realization of this paper are available in the paper itself and on the GenBank database.

## Supporting Information

Supporting Information Figure S1. Bootstrap consensus tree inferred from 1000 replicates realized with MEGA software. The nucleotide substitution model (K2 + G) was chosen after a BIC-score analysis. The percentages of replicate trees in which the associated taxa clustered together in the bootstrap analysis are shown next to the internal branches. Each color corresponds to a specific set of sequences, as indicated by the colored squares under the tree. Table S1. Accession numbers, host, geographic origin, and strain information of *L. tarentolae*, *Leishmania* sp., *Leishmania adleri*, and outgroups ITS-1 sequences included in the phylogenetic analysis. Chinese sequences (not shown) have been obtained from the datasets of the following publications: 10.1016/j.actatropica.2016.06.023; https://doi.org/10.1371/journal.pone.0210681; 10.1007/s00436-010-1969-9. Table S2. Accession numbers, host, and source of the *L. tarentolae* ITS-1 sequences generated in the study.
