# Supplementary material for: Sand Fly Fauna and Prevalence of Leishmania spp. in a Newly Investigated Area of Northern Italy: Emerging Epidemiological Scenarios?
Source: Transbound Emerg Dis. 2025 Jul 21;2025:4426385. doi: 10.1155/tbed/4426385 (PMC12303632; doi:10.1155/tbed/4426385)
Supplement: Supporting Information — Figure S1. Bootstrap consensus tree inferred from 1000 replicates realized with MEGA software. The nucleotide substitution model (K2 + G) was chosen after a BIC-score analysis. The percentages of replicate trees in which the associated taxa clustered together in the bootstrap analysis are shown next to the internal branches. Each color corresponds to a specific set of sequences, as indicated by the colored squares under the tree. Table S1. Accession numbers, host, geographic origin, and strain information of L. tarentolae, Leishmania sp., Leishmania adleri, and outgroups ITS-1 sequences included in the phylogenetic analysis. Chinese sequences (not shown) have been obtained from the datasets of the following publications: 10.1016/j.actatropica.2016.06.023; https://doi.org/10.1371/journal.pone.0210681; 10.1007/s00436-010-1969-9. Table S2. Accession numbers, host, and source of the L. tarentolae ITS-1 sequences generated in the study. [file 4426385.f1.docx]

**Supplementary material**


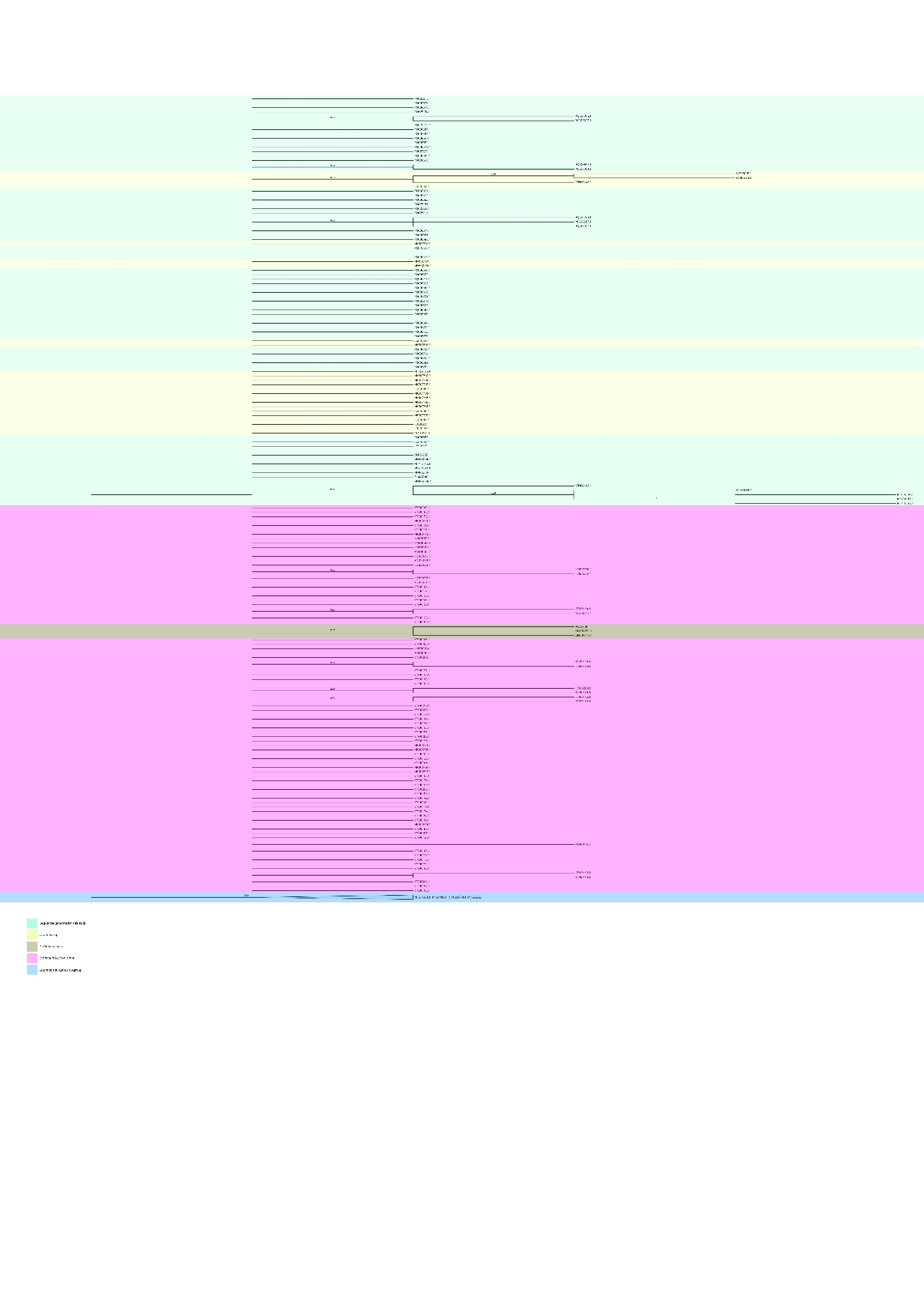


**Supplementary figure 1.** Bootstrap consensus tree inferred from 1000 replicates realized with MEGA software. The nucleotide substitution model (K2 + G) was chosen after a BIC-score analysis. The percentage of replicate trees in which the associated taxa clustered together in the bootstrap test 1000 replicates are shown next to internal branches. Each colour corresponds to a specific set of sequences, as indicated by the coloured squares under the tree.

| **Accession number** | **Species** | **Host** | **Origin** | **Strain/isolate** |
| --- | --- | --- | --- | --- |
| MW930737.1 | *L. tarentolae* | Human | Italy - Pelagie | LIU55 |
| LC086293.1 | *L. tarentolae* | *S. minuta* | Spain - Extremadura | / |
| MT416149.1 | *L. tarentolae* | Human | Italy - Lazio | / |
| MW930737.1 | *L. tarentolae* | Human | Italy - Pelagie | / |
| KX035099.1 | *L. tarentolae* | *S. dentata* | Turkey | / |
| MW930736.1 | *L. tarentolae* | Human | Italy - Pelagie | / |
| MW832546.1 | *L. tarentolae* | *Canis lupus familiaris* | Italy - Apulia | / |
| CP119851.1 | *L. tarentolae* | */* | Seattle - Lab-maintained | M2 |
| MW832548.1 | *L. tarentolae* | *S. minuta* | Italy - Apulia | / |
| OL449848.1 | *L. tarentolae* | *T. mauritanica* | Italy - Apulia, Calabria, Sicily | Leishtar4 |
| MW832547.1 | *L. tarentolae* | *P. siculus* | Italy - Apulia | isolate 2 |
| MT416146.1 | *L. tarentolae* | Human | Italy - Lazio | isolate 362 |
| MT416142.1 | *L. tarentolae* | Human | Italy - Lazio | isolate 389 |
| MT416143.1 | *L. tarentolae* | Human | Italy - Lazio | isolate 764 |
| MT416145.1 | *L. tarentolae* | Human | Italy - Lazio | isolate 868 |
| MT416144.1 | *L. tarentolae* | Human | Italy - Lazio | isolate 862 |
| MT416141.1 | *L. tarentolae* | Human | Italy - Lazio | isolate 309 |
| MT416147.1 | *L. tarentolae* | Human | Italy - Lazio | isolate 970 |
| KU680858.1 | *L. tarentolae* | */* | Tehran - Lab-maintained | P10 |
| LC028233.1 | *Leishmania* sp. | *S. minuta* | Portugal | / |
| LC216356.1 | *Leishmania* sp. | *S. minuta* | Spain - Madrid | / |
| MK567806 | *Leishmania* sp. | *S. minuta* | Spain - Madrid | / |
| MT302155.1 | *Leishmania* sp. | *Canis lupus familiaris* | Iran | / |
| MK567786.1 | *Leishmania* sp. | *S. minuta* | Spain - Madrid | / |
| MK567797.1 | *Leishmania* sp. | *S. minuta* | Spain - Madrid | / |
| LC216369.1 | *Leishmania* sp. | *S. minuta* | Spain - Madrid | / |
| MK567789.1 | *Leishmania* sp. | *S. minuta* | Spain - Madrid | / |
| MK567790.1 | *Leishmania* sp. | *S. minuta* | Spain - Madrid | / |
| MK567785.1 | *Leishmania* sp. | *S. minuta* | Spain - Madrid | / |
| MK567794.1 | *Leishmania* sp. | *S. minuta* | Spain - Madrid | / |
| MK567792.1 | *Leishmania* sp. | *S. minuta* | Spain - Madrid | / |
| MK567788.1 | *Leishmania* sp. | *S. minuta* | Spain - Madrid | / |
| LC216363.1 | *Leishmania* sp. | *S. minuta* | Spain - Madrid | / |
| LC216358.1 | *Leishmania* sp. | *S. minuta* | Spain - Madrid | / |
| LC028235.1 | *Leishmania* sp. | *S. minuta* | Portugal | / |
| LC216360.1 | *Leishmania* sp. | *S. minuta* | Spain - Madrid | / |
| MK567795.1 | *Leishmania* sp. | *S. minuta* | Spain - Madrid | / |
| LC216367.1 | *Leishmania* sp. | *S. minuta* | Spain - Madrid | / |
| AJ300480.1 | *Leishmania adleri* | */* | Russia | ISEG/SU/72/A7 |
| ON731776.1 | *Leishmania adleri* | *Sergentomyia clydei* | Kenya | / |
| OQ304332.1 | *Leishmania adleri* | *Sergentomyia sp.* | Niger | / |
| MF597933.1 | *Leishmania infantum* | *P. perniciosus* | Tunisia | / |
| MN244151.1 | *Leishmania donovani* | *A. fuscipes* | Senegal | / |
| KX821679.1 | *Leishmania major* | *H. sapiens* | Austria | MHOM/AT/16/AS16 |
| MF926263.1 | *Leishmania tropica* | *H. sapiens* | Syria | / |

**Supplementary table 1.** Accession numbers, host, geographic origin and strain information of *L. tarentolae*, *Leishmania* sp., *Leishmania adleri*, and outgroups ITS-1 sequences included in the phylogenetic analysis. Chinese sequences (not shown), have been obtained from the datasets of the following publications: 10.1016/j.actatropica.2016.06.023; https://doi.org/10.1371/journal.pone.0210681; 10.1007/s00436-010-1969-9.

| **Accession_Number** | **Host** | **Source** |
| --- | --- | --- |
| PQ608673.1 | *Sergentomyia minuta* | Whole sand fly |
| PQ608674.1 | *Sergentomyia minuta* | Whole sand fly |
| PQ608676.1 | *Sergentomyia minuta* | Whole sand fly |
| PQ608677.1 | *Sergentomyia minuta* | Whole sand fly |
| PQ608681.1 | *Sergentomyia minuta* | Whole sand fly |
| PQ608682.1 | *Sergentomyia minuta* | Whole sand fly |
| PQ608683.1 | *Sergentomyia minuta* | Whole sand fly |
| PQ608685.1 | *Sergentomyia minuta* | Whole sand fly |
| PQ608687.1 | *Sergentomyia minuta* | Whole sand fly |
| PQ608690.1 | *Sergentomyia minuta* | Whole sand fly |
| PQ608692.1 | *Sergentomyia minuta* | Whole sand fly |
| PQ608693.1 | *Sergentomyia minuta* | Whole sand fly |
| PQ608695.1 | *Sergentomyia minuta* | Whole sand fly |
| PQ608702.1 | *Sergentomyia minuta* | Whole sand fly |
| PQ608703.1 | *Sergentomyia minuta* | Whole sand fly |
| PQ608704.1 | *Sergentomyia minuta* | Whole sand fly |
| PQ608707.1 | *Sergentomyia minuta* | Whole sand fly |
| PQ608708.1 | *Sergentomyia minuta* | Whole sand fly |
| PQ625219.1 | *Sergentomyia minuta* | Whole sand fly |
| PQ625221.1 | *Sergentomyia minuta* | Whole sand fly |
| PQ608679.1 | *Phlebotomus perniciosus* | Whole sand fly |
| PQ608680.1 | *Phlebotomus perniciosus* | Whole sand fly |
| PQ608686.1 | *Phlebotomus perniciosus* | Whole sand fly |
| PQ608697.1 | *Phlebotomus perniciosus* | Whole sand fly |
| PQ608698.1 | *Phlebotomus perniciosus* | Whole sand fly |
| PQ608699.1 | *Phlebotomus perniciosus* | Whole sand fly |
| PQ608700.1 | *Phlebotomus perniciosus* | Whole sand fly |
| PQ608709.1 | *Phlebotomus perniciosus* | Whole sand fly |
| PQ625215.1 | *Phlebotomus perniciosus* | Whole sand fly |
| PQ625217.1 | *Phlebotomus perniciosus* | Whole sand fly |
| PQ625222.1 | *Phlebotomus perniciosus* | Whole sand fly |
| PQ625223.1 | *Phlebotomus perniciosus* | Whole sand fly |
| PQ625225.1 | *Phlebotomus perniciosus* | Whole sand fly |
| PQ608684.1 | *Phlebotomus neglectus* | Whole sand fly |
| PQ625220.1 | *Phlebotomus neglectus* | Whole sand fly |
| PQ608675.1 | *Podarcis muralis* | Blood |
| PQ608678.1 | *Podarcis muralis* | Blood |
| PQ608688.1 | *Podarcis muralis* | Faeces |
| PQ608689.1 | *Podarcis muralis* | Faeces |
| PQ608691.1 | *Podarcis muralis* | Tail |
| PQ608694.1 | *Podarcis muralis* | Cloacal swab |
| PQ608696.1 | *Podarcis muralis* | Oral swab |
| PQ608701.1 | *Podarcis muralis* | Oral swab |
| PQ608705.1 | *Podarcis muralis* | Oral swab |
| PQ608706.1 | *Podarcis muralis* | Faeces |
| PQ625216.1 | *Podarcis muralis* | Cloacal swab |
| PQ625218.1 | *Podarcis muralis* | Faeces |
| PQ625224.1 | *Podarcis muralis* | Faeces |
| PQ625226.1 | *Podarcis muralis* | Blood |
| PQ625227.1 | *Podarcis muralis* | Blood |
| PQ686310.1 | *Leishmania tarentolae* strain P10 | Lab colture |

**Supplementary table 2.** Accession numbers, host, and source of the *L. tarentolae* ITS-1 sequences generated in the study.
